# Specific, Surface-Driven, and High-Affinity Interactions of Fluorescent Hyaluronan with PEGylated Nanomaterials

**DOI:** 10.1021/acsami.9b17974

**Published:** 2020-01-29

**Authors:** Francesco Palomba, Enrico Rampazzo, Nelsi Zaccheroni, Marco Malferrari, Stefania Rapino, Valentina Greco, Cristina Satriano, Damiano Genovese, Luca Prodi

**Affiliations:** † Dipartimento di Chimica “Giacomo Ciamician”, Università di Bologna, via Selmi 2, 40126 Bologna, Italy; ‡ Consorzio Interuniversitario di Ricerca in Chimica dei Metalli nei Sistemi Biologici (C.I.R.C.M.S.B.), via Celso Ulpiani, 27, 70125 Bari, Italy; § Dipartimento di Scienze Chimiche, Università degli Studi di Catania, viale Andrea Doria 6, 95125 Catania, Italy

**Keywords:** hyaluronic acid, fluorescence, nanomaterial, super-resolution, cell internalization

## Abstract

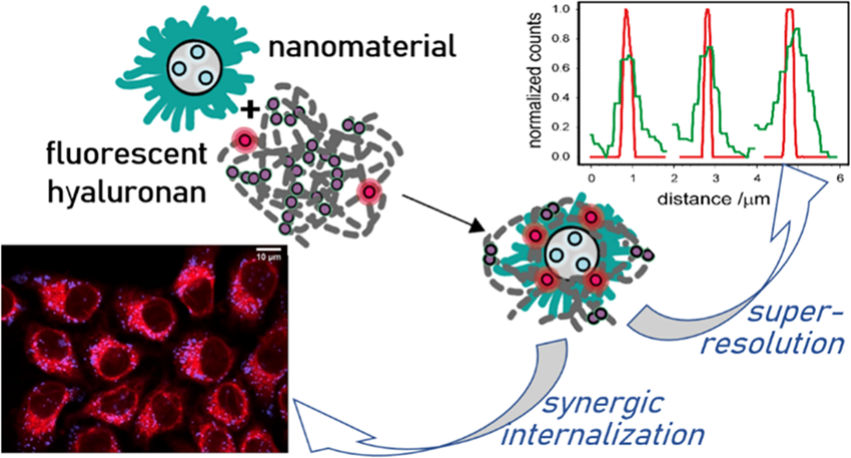

Hybrid nanomaterials are a subject of extensive research in nanomedicine, and their clinical application is reasonably envisaged in the near future. However, the fate of nanomaterials in biological environments poses serious limitations to their application; therefore, schemes to monitor them and gain control on their toxicity could be of great help for the development of the field. Here, we propose a probe for PEGylated nanosurfaces based on hyaluronic acid (HA) functionalized with rhodamine B (RB). We show that the high-affinity interaction of this fluorogenic hyaluronan (HA-RB) with nanoparticles exposing PEGylated surfaces results in their sensing, labeling for super-resolution imaging, and synergistic cellular internalization. HA-RB forms nanogels that interact with high affinity—down to the picomolar range—with silica nanoparticles, selectively when their surface is covered by a soft and amphiphilic layer. This surface-driven interaction triggers the enhancement of the luminescence intensity of the dyes, otherwise self-quenched in HA-RB nanogels. The sensitive labeling of specific nanosurfaces also allowed us to obtain their super-resolution imaging via binding-activated localization microscopy (BALM). Finally, we show how this high-affinity interaction activates a synergistic cellular uptake of silica nanoparticles and HA-RB nanogels, followed by a differential fate of the two partner nanomaterials inside cells.

## Introduction

Nanomaterials tested for medical purposes feature a broad diversity of compositions, architectures, surfaces, shapes and dimensions, a variety that not only enriches the portfolio of strategies^1^ but also makes difficult a clear definition of their fate in biological and natural environments and that could consequently obstacle their application in these fields.^2,3^ The surface of nanomaterials, in particular, plays a key role in determining the interactions of a nano-object with its environment, resulting in specific properties in terms of biodistribution, circulation time, bioaccumulation, and targeting.^1^ Among the many approaches to control the surface chemistry of nanomaterials, PEGylation has emerged as most promising due to strong colloidal stabilization and the ability to shield nanomaterials from the formation of protein coronas, resulting in prolonged circulation time, higher probability of reaching the target, lower undesired accumulation, and lower toxicity.^4−7^

Hyaluronic acid (HA), a major component of cell-surface glycoproteins and of the extracellular matrix, is already largely used in cosmetic and pharmaceutical industries and owing to its high biocompatibility, ease of manipulation and use, it is also drawing increasing attention in nanomedicine.^8−10^ As a polymer and a polyelectrolyte, it is subject to multivalent interactions with (nano)surfaces both in its pristine state and upon functionalization.^11^ At the cellular and subcellular level, the special mix of H-bonding properties, high molecular weight, and hydrophobic residues, which can be organized in hydrophobic patches and pockets, endow hyaluronan-based nanostructures with marked drug delivery ability, based on either covalent or noncovalent interactions.^12−14^ Also, in this case, there are elegant examples of improved targeting and prolonged circulation time of hyaluronic acid-based nanocarriers involving their PEGylation.^15,16^

The unique characteristics of HA also allow for its active role in interfacing cells with external agents. It was recently found, for example, that a set of proteins involved in hyaluronan binding also forms fingerprint coronas on gold and silver nanoparticles (NPs) and may hence act as bridges that mediate the interaction of nanoparticles with cellular surfaces.^17−19^ HA has also been reported to interact with micelles of oppositely charged sugar-based surfactants^20^ and with quantum dots covered with a positively charged polymeric shell.^21^ Exogenous nanomaterials, including pollutants, might be recognized as a function of their size and surface properties, such as hydrophilicity, hardness, and roughness.

The hydrophobic acetyl residues play an active role in CD44 transmembrane glycoprotein recognition,^22,23^ which occurs through a robust interaction, only affected by heavy structural modifications of HA.^24^ Hydrophobicity of HA can in particular emerge upon special treatments of HA (e.g., freeze-drying^25^) or upon specific functionalization.^26^ The mix of hydrophobic patches and hydrophilic moieties allows, upon functionalization with proper dyes, to create nanostructured particles and gels in which fluorescent moieties are mainly aggregated and that, most interestingly, can be disaggregated in response to specific stimuli, in particular, under the action of HAase.^10,27−29^

Here, we take advantage of these findings to design a sensitive probe for PEGylated nanosurfaces based on HA: upon straightforward functionalization with rhodamine B (RB) isothiocyanate dyes, we obtain nanogels (HA-RB) in which RB dyes suffer from self-quenching due to aggregation. HA-RB nanogels can efficiently interact with pegylated layers on nanomaterial surfaces, overcoming the small driving force of rhodamine B toward aggregation. This interaction occurs in water, in a large range of pH and ionic strength, and drives the partial disentanglement of RB-functionalized HA nanogels leading to a substantial fluorescence enhancement (up to 10 times). In addition, the resulting assemblies can modify the interaction of both HA and the nanomaterials with other biostructures. The results presented here indicate that the internalization process of the components is indeed modified in the assembly in a synergic-like way, that could be ideally exploited to improve, for example, drug delivery.

## Results and Discussion

**Figure 1:**
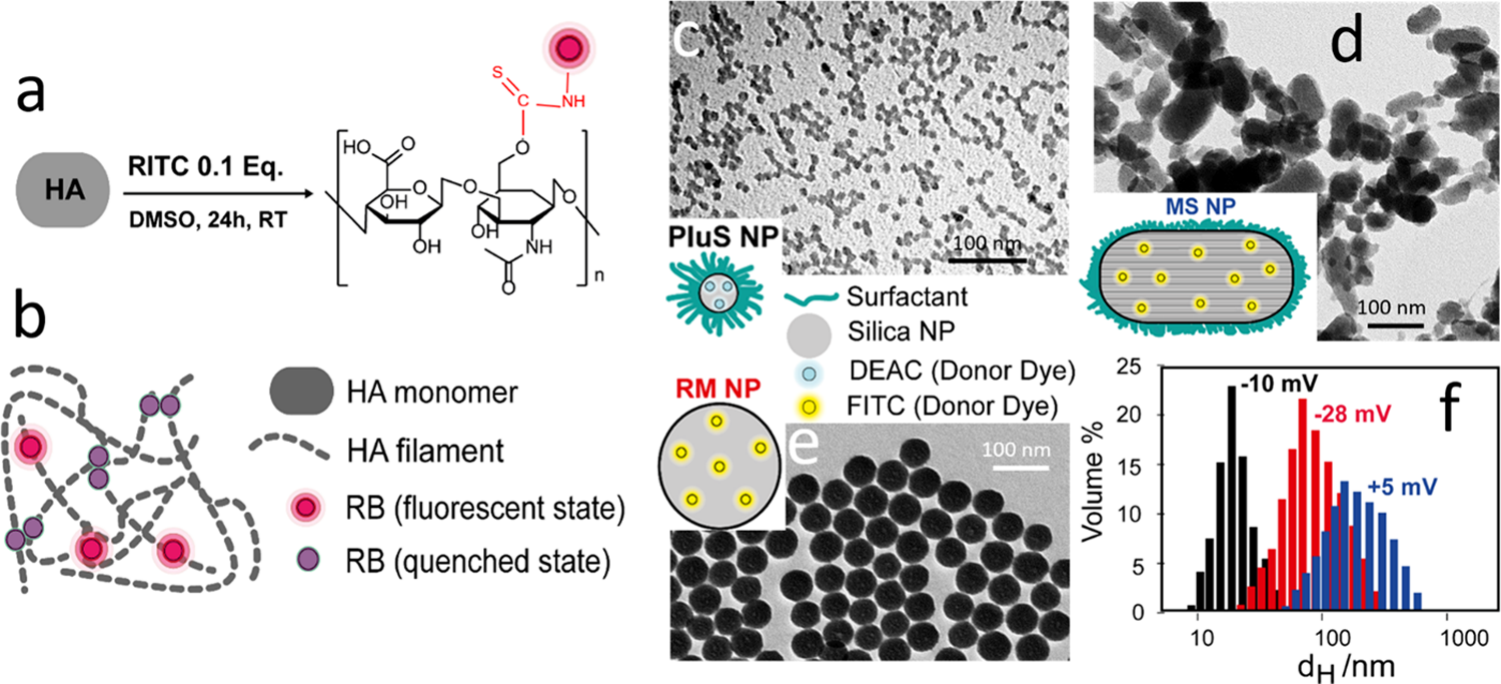
(a) Reaction scheme of HA with RITC (RB) to yield HA-RB with thiocarbamate bond formation. (b) Cartoon of aggregated HA-RB, with RB units in fluorescent and quenched states. (c) Cartoon and transmission electron microscopy (TEM) image of PluS NPs doped with diethylaminocoumarine (DEAC). (d) Cartoon and TEM image of MS NPs doped with fluorescein. (e) Cartoon and TEM image of RM NPs doped with fluorescein. (f) Hydrodynamic diameter distributions obtained by dynamic light scattering (DLS) (volume distribution, phosphate-buffered saline (PBS)) with corresponding average ζ-potentials (black: PluS NP; red: RM NP; blue: MS NP).

Synthesis and characterization of HA-RB nanogels. HA-RB nanogels are obtained via reaction of hyaluronic acid (193 ± 2 kDa) with rhodamine B isothiocyanate (RITC) in dimethyl sulfoxide (DMSO), which proceeds through nucleophilic attack by the methyl-hydroxyl group of the polysaccharide to the reactive isothiocyanate groups, as reported previously for similar polymers (Figure 1a).^30^ The initially insoluble HA is progressively dispersed during the reaction in the DMSO solvent, which also concurs to the effectiveness of the nucleophilic attack^31^ and a homogeneous transparent dispersion is obtained after a few hours. The reaction proceeds for 24 h at room temperature, and the resulting dispersion is dialyzed against water, to obtain a nonturbid, concentrated, aqueous dispersion of HA-RB nanogels. After work-up, the effective dye doping degree was evaluated by ultraviolet–visible (UV–vis) analysis measuring the absorbance of rhodamine B, while the weight of the product matched with the starting amount of HA, indicating that no significant loss of materials occurred during synthesis and work-up, except for the unreacted RB. An effective derivatization degree amounted to 1 fluorophore every 25 HA repeat units, i.e., about 19 dyes per polymer chain.

HA-RB nanogels at 100 nM concentration in PBS feature hydrodynamic diameter *d*_H_ = 150 ± 35 nm with PdI = 0.255 (Figure S7, from DLS measurements) and a negative ζ-potential of −17 ± 2 mV. The absorption spectrum of HA-RB in water reveals a low value of the peak over shoulder ratio (*A*_p_/*A*_s_ = 1.21, Table 1), indicating that RB dyes are heavily aggregated at the ground state. The aggregation is also confirmed by the very low emission quantum yield (0.019) compared that of RB in water (0.30). The observed monoexponential decay (τ_average_ = 1.58 ns) of HA-RB indicates—since it is very similar to that observed for nonaggregated RB in water (1.68 ns)—that the small emission signal observed can be assigned to a minority of RB dyes not suffering from heavy quenching, while most RB dyes are completely quenched and thus nonemissive, as schematized in Figure 1b. Furthermore, even the rather high emission anisotropy (*r* = 0.10 ± 0.02) is in agreement with these assumptions, indicating that the emissive RB dyes are bound to the nanogels and are not freely diffusing in water.

**Table 1:** Photophysical Data of HA-RB in Different Solvents and in Presence of Different Nanomaterials, before and after Calcination^a^

	*A*_p_/*A*_s_^b^		Φ^c^		τ/ns^d^	
ethanol	2		0.23		3.07	
water	1.21		0.019		1.58	
	pristine	calcined	pristine	calcined	pristine	calcined
PluS NPs	1.76	1.33	0.083	0.023	3.18	1.59
MS NPs	1.85	1.55	0.095	0.022	2.28	1.58
RM NPs	1.45	1.40	0.021	0.020	1.59	1.59

a [HA-RB] = 200 nM (or 0.039 mg/mL), NP concentration = 0.055 mg/mL.

b Ratio of absorbance at peak (560 nm, *A*_p_) and shoulder (530 nm, *A*_s_).

c Fluorescence quantum yield.

d Fluorescence lifetime.

Native HA is extremely soluble in water and rather insoluble in ethanol. Surprisingly, when dissolved in ethanol, HA-RB becomes strongly emissive and its absorption spectrum, fluorescence quantum yield, and lifetime (Table 1) indicate that RB dyes are not interacting with each other. When a small amount of HA-RB in ethanol is redispersed in water, the RB dyes exhibit again the same absorbance and emission spectra typical of quenched aggregates. Increasing the water:ethanol ratio, the photophysics of RB dyes reveals that HA-RB progressively reaggregates in the aqueous environment (Figure S9). This finding further confirms that RB dyes are covalently linked to HA, since after being well dissolved and emissive in ethanol they aggregate and quench again as soon as water is added to the system, even at low concentration ([HA-RB] = 200 nM, [RB dyes] = 3.8 μM).

### Interaction of HA-RB Nanogels with Silica Nanoparticles According to Their Surfaces

A set of silica-based nanoparticles was designed and prepared to investigate the ability of HA-RB to interact with nanomaterials, depending on their size and on the chemistry of their surrounding shell, to shed light on the occurring interaction at the interface. HA-RB nanogels have been tested in PBS versus increasing amounts of three types of nanoparticle (NP) suspensions: (i) Pluronic-silica (PluS) nanoparticles,^32,33^ (ii) mesoporous silica (MS) nanoparticles, and (iii) reverse microemulsion (RM) nanoparticles, whose cartoons are depicted in Figure 1c–e, while the synthesis is described in the Supporting Information. PluS NPs are very small micelle-like nanoparticles, with a core–shell morphology featuring a silica core of 11 nm diameter and a poly(ethylene glycol) (PEG) shell with a total hydrodynamic diameter of 25 nm (Figure S7) due to the Pluronic F127 surfactant used during the template synthesis and a ζ-potential of −10 ± 4 mV. MS NPs are much larger (160 nm as their average diameter) but with similar surface area and coverage, since the surfactants CTAB and Pluronic F127 used during their preparation are not calcined post synthesis, with a final ζ-potential of +5 ± 3 mV. Finally, RM NPs are bare silica nanoparticles of an intermediate diameter of 55 nm and a different exposed surface, since the short TritonX-100 surfactant molecules used in the reverse microemulsion are efficiently removed in the work-up phase by repeated washings with acetone, ethanol and then water, yielding a bare silica surface with a ζ-potential of −28 ± 7 mV (Figure 1f).

**Figure 2:**
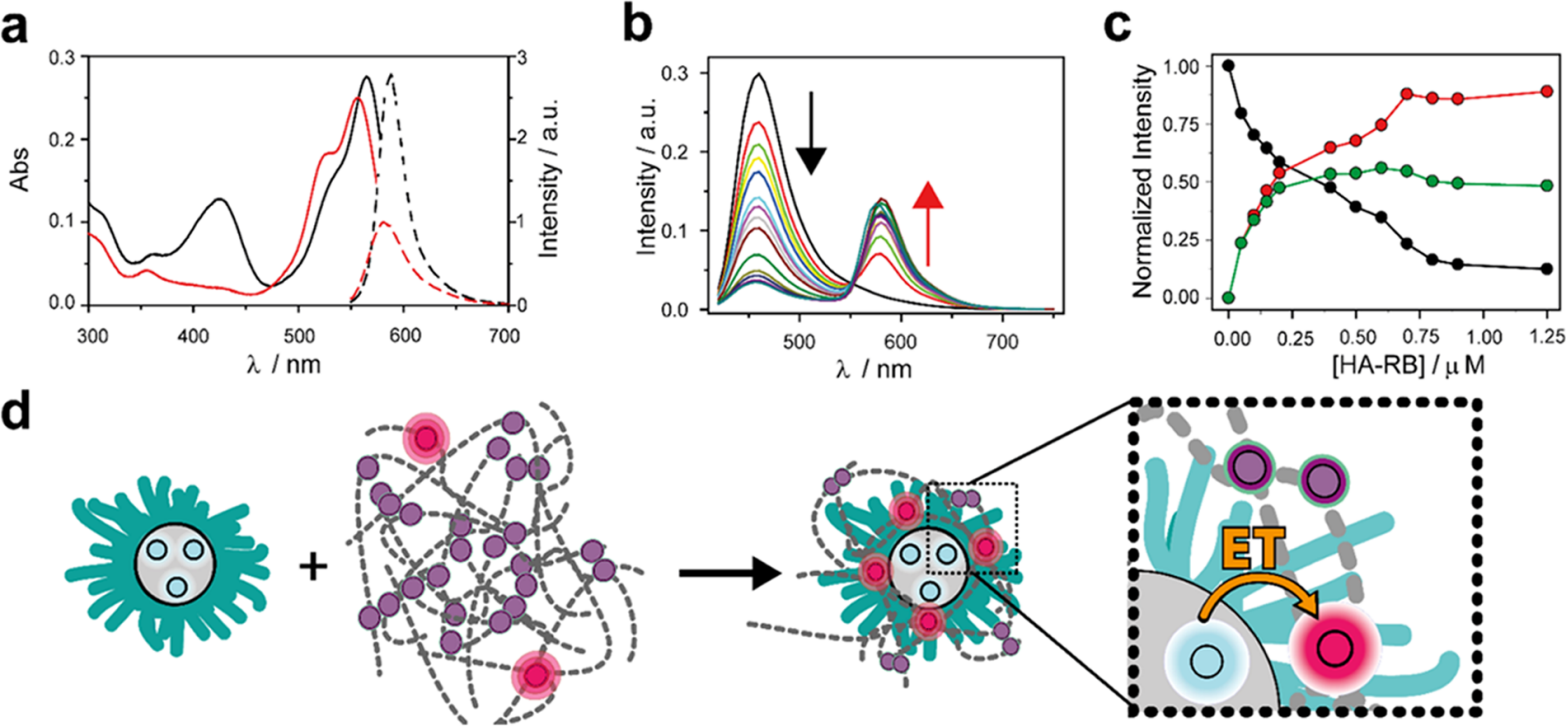
Photophysical and Forster resonance energy transfer (FRET) study of HA-RB in presence of DEAC-doped PluS NPs. (a) Absorbance (solid lines) and emission (dashed lines, λ_exc_ = 530 nm) spectra of HA-RB 0.2 μM in PBS before (red lines) and after addition of 1 μM DEAC-doped PluS NPs (black lines). (b) Emission spectra (λ_exc_ = 400 nm) of DEAC-doped PluS NPs (200 nM) upon addition of increasing amounts of HA-RB (0–1.25 μM). (c) Trends of the titration in (b): DEAC-doped PluS NP emission (black circles, quenching of energy donors, λ_exc_ = 400 nm, λ_em_ = 455 nm), HA-RB (λ_em_ = 580 nm, directly excited at λ_exc_ = 530 nm, red circles, or as energy acceptor at λ_exc_ = 400 nm—green circles). All fluorescence data are corrected for the instrumental calibration curve and inner filter effects. (d) Cartoon of the interaction-driven energy transfer (ET) from DEAC-doped PluS NPs to HA-RB.

When increasing amounts HA-RB nanogels are added to a PBS solution of PluS NPs, the photophysics of RB dyes in the HA-RB nanogels displays important variations, with a remarkable change of the absorption spectrum and the increase of fluorescence intensity. The change of the absorption spectrum (Figures S3 and S4) mainly concerns the probabilities associated to the vibronic bands of the S_0_ → S_1_ transition and can be quantitatively monitored by the absorbance ratio of the peak (*A*_p_ at 560 nm) to shoulder (*A*_s_ at 530 nm). At the same time, fluorescence intensity (Figure 2a) shows a large increase, from 0.019 to 0.083 (Table 1), of the average fluorescence quantum yield (Φ) of RB dyes in HA-RB, in the presence of PluS NPs. Finally, also the fluorescence lifetime of HA-RB increases from 1.59 to 3.18 ns, which is typical of RB in nonaqueous environments. Overall, the changes of the photophysical parameters considered, i.e., *A*_p_/*A*_s_, Φ, and τ, indicate that a significant disaggregation, although not complete, as it can be observed by the incomplete recovery of *A*_p_/*A*_s_ and of Φ, of RB dyes occurs in presence of PluS NPs (Table 1). Disaggregation stems from a favorable interaction with these NPs that promote the partial disentanglement of HA-RB nanogels (forming an aggregate of an average diameter of 71 nm, see Figure S7), owing to the NPs small size and the surface chemistry dominated by the PEG branches, in which part of the RB moieties can interdigitate, finding a less polar environment. Notably, PluS NPs have actually been recently proven to feature complex nanoenvironments in their shell, with a strong polarity gradient.^34^

To obtain an additional direct evidence of the interaction between HA-RB and PluS NPs, we doped this kind of NPs with a donor dye (a diethylaminocoumarine derivative, DEAC) and we monitored the occurrence of an energy transfer process from nanoparticles to HA-RB, where rhodamine B could act as the acceptor dye. On the basis of their photophysical properties, the energy transfer process occurs when energy donor and acceptors are closer than approximately 8 nm (the Förster Radius for DEAC and rhodamine B is 3.8 nm) inducing the quenching of the donor emission and the simultaneous sensitization of the acceptor one. We monitored the occurrence of this process; as shown in Figure 2b,c, when HA-RB is added (0–1.25 μM) to a 200 nM PluS NP dispersion, the DEAC fluorescence is strongly quenched, reaching a quenching efficiency higher than 80% upon addition of 4 equiv of HA-RB. At the same time, a corresponding sensitized HA-RB emission can be observed at 585 nm upon excitation at 400 nm, where the absorption of the DEAC dyes is by far prevalent. Interestingly, the sensitized emission intensity of RB increases only until 1 equiv of HA-RB is added; then, it reaches a plateau and finally undergoes a slight decrease when more than 3 equiv of HA-RB is added. Directly excited (nonsensitized) HA-RB emission was also measured upon excitation at 530 nm.

As shown in Figure 2c, in this case, the intensity increases in all of the concentration range observed but with a different slope. This slope is particularly meaningful, since it is proportional to the fluorescence quantum yield of the added aliquots of RB; in fact, the higher the slope, the higher is the fluorescence increase at the same variation of concentration (and thus absorbance). In particular, we think that the obtained results reasonably indicate that the first equivalent of HA-RB wraps the NPs up forming a first layer around the nanostructures, inducing the already discussed large increase of the fluorescence intensity while, at higher concentration, the newly added RB moieties show a quenched fluorescence. However, the fact that the quenching efficiency of the DEAC increases even after the formation of a first layer of HA suggests that the polymer could form additional layers still close enough to the NPs to quench the DEAC fluorescence but presenting a too low disentanglement degree to decrease their self-quenching processes. This hypothesis is also in agreement with the sensitized emission trend; in fact, keeping in mind that in this case, the absorbance increase at the excitation wavelength is negligible, we observe that the formation of the first layer leads to a quite efficient energy transfer toward highly emitting dyes, while an additional amount of HA-RB leads to the sensitization of poorly emitting dyes (Figure 2d).

**Figure 3:**
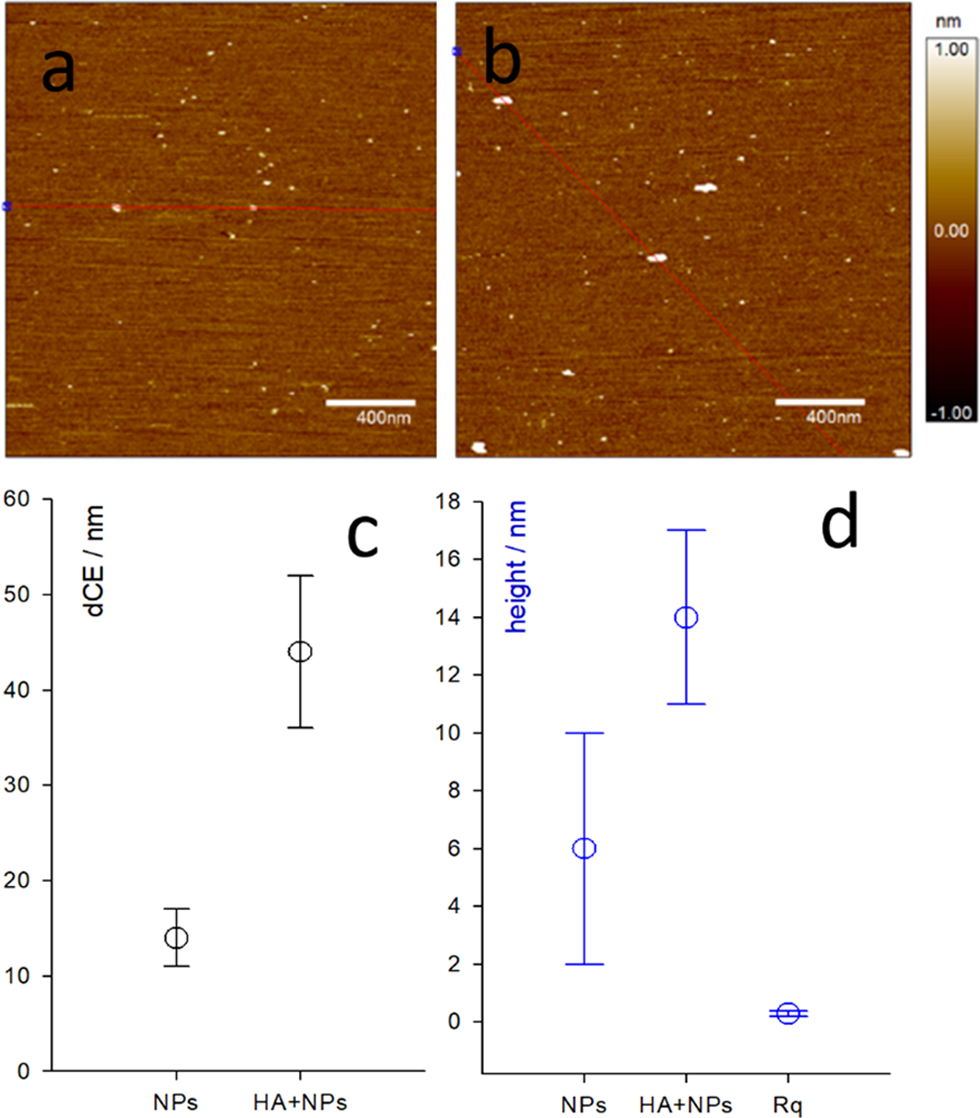
AFM topography images recorded in water of PluS NPs (a) and PluS NPs + HA-RB (b, 1:1 mol ratio). (c, d) The average equivalent circle diameters and the average height of features in figures (a) and (b) are plotted in panels (c) and (d), respectively.

Atomic force microscopy (AFM) analyses provide further evidence of the interaction between HA-RB and PluS NPs (Figure 3). Indeed, the height micrographs of the samples imaged in water clearly show the size increase of Plus NPs upon association with HA-RB, with average values in the circle equivalent diameter (*d*_CE_, i.e., the diameter of a circle with the same area as the two-dimensional (2D) image of the particle) and the maximum height (*Z*_max_, i.e., the greatest *z* value found on the particle) of *d*_CE_ = 14 ± 3 nm and *Z*_max_ = 6 ± 4 nm for the nanoparticles alone, and *d*_CE_ = 44 ± 8 nm and *Z*_max_ = 14 ± 3 nm for the polymer–nanoparticle hybrids, respectively. The section analysis profiles evidence the wrapping of the nanoparticles by the hyaluronan polymer chains, resulting in a core–shell structure. The AFM imaging of the samples in air (see Figure S8) also confirms the effective coating of the NPs by HA-RB as well as a disaggregation effect of HA-RB by the nanoparticles.

**Figure 4:**
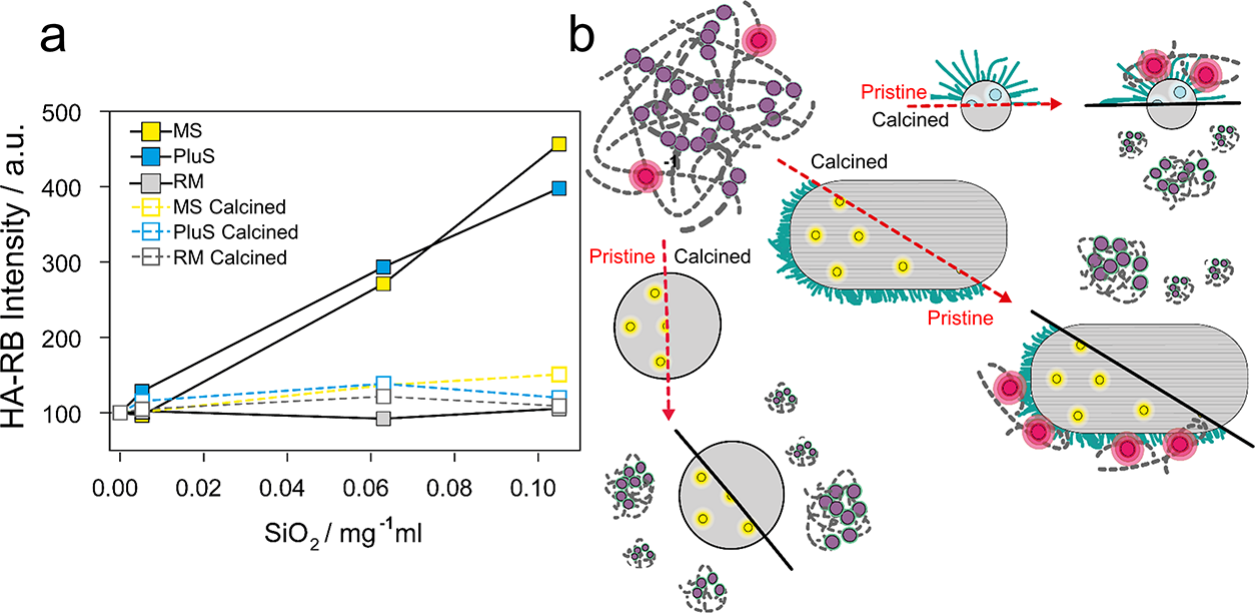
(a) Normalized emission intensity of HA-RB in presence of increasing amounts of MS (yellow), PluS (cyan), and RM NPs (gray), before (full squares) and after calcination (empty squares). (b) Cartoon of the interaction between HA-RB and the various silica NPs before and after calcination.

MS NPs yielded similar effects as PluS NPs on the photophysical properties of HA-RB, increasing both its *A*_p_/*A*_s_ and Φ (Table 1 and Figure 4). We used FITC-functionalized MS NPs to follow FRET from the NPs to HA-RB: we observed efficient quenching of FITC (80% as shown in Figure S2) and, also in this case, the fluorescence lifetime of RB dyes increases to 2.28 (Table 1).

On the contrary, upon addition of HA-RB to a dispersion of RM NPs (doped with FITC for FRET experiments), we did not observe any quenching of the donor dyes, and only a very minor effect on *A*_p_/*A*_s_ and Φ of RB (Table 1 and Figure 4), indicating a negligible interaction of this kind of nanoparticles with HA-RB. All of these findings demonstrate a significant specificity of HA-RB in the recognition of nanostructured surfaces bearing PEGylated shells.

To further investigate this point, the same experiments were performed with PluS NPs and mesoporous NPs after calcination. Calcination eliminates the soft, amphiphilic shell from the NP surface (and also all organic molecules, including dyes embedded in NPs). Therefore, the NPs tested in this experiment are made of bare silica, without organic counterparts, and characterized by less colloidal stability due to the absence of the amphiphilic stabilizing shell. In these cases, their presence does not induce relevant changes in either *A*_p_/*A*_s_ and Φ of HA-RB in water. As evident in Figure 4a, after calcination, all nanomaterials are ineffective in interacting with HA-RB and in enhancing its luminescence, proving once more that naked nanostructured silica is not able to disentangle the nanogels where RB dyes are self-quenched by aggregation (as schematized in Figure 4b).

### Proofing Super-Resolution Imaging: High-Affinity Interaction of HA-RB with Surface-Bound PluS NPs

**Figure 5:**
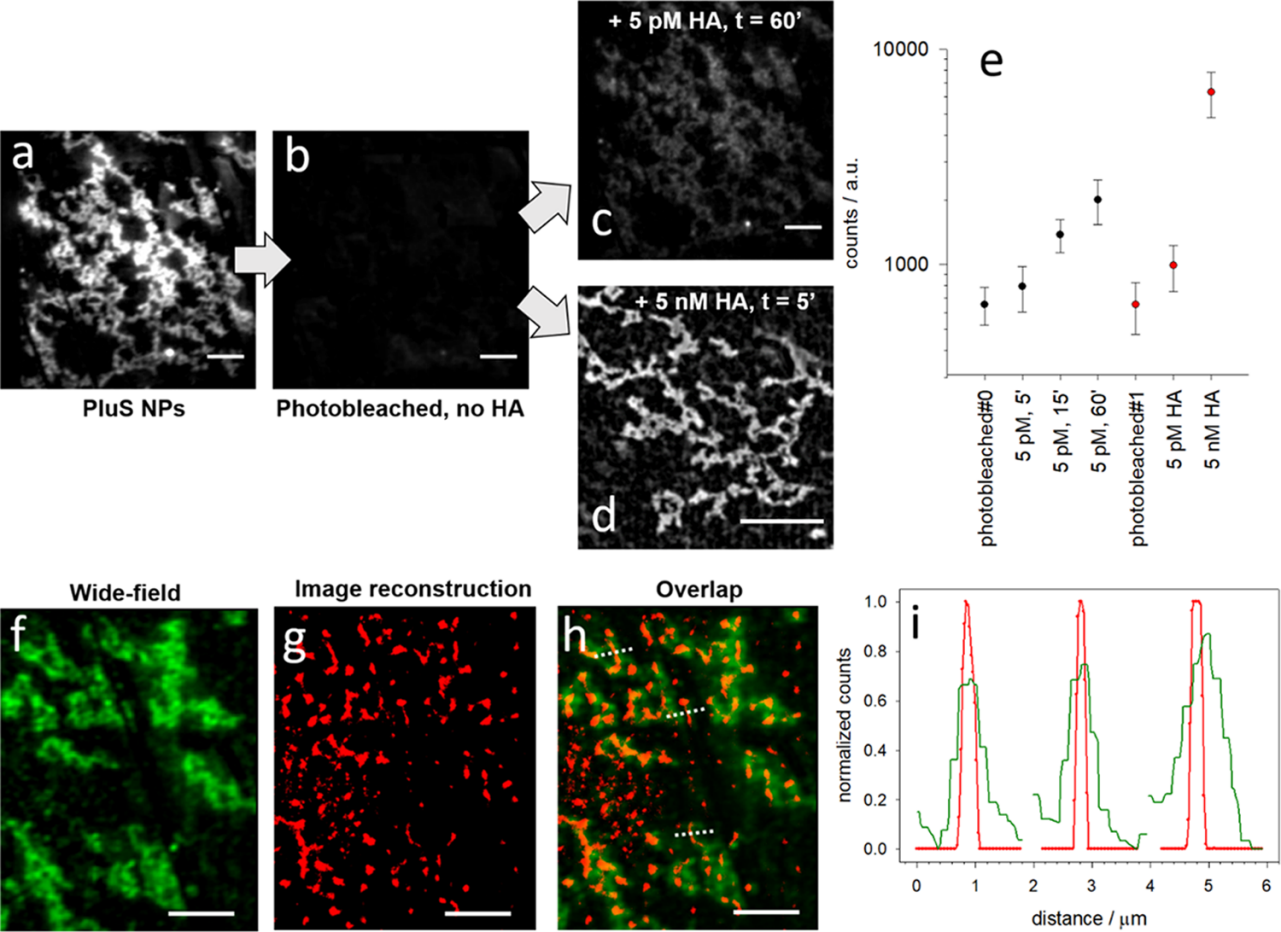
Wide-field fluorescence microscopy images of a coverglass covalently functionalized with PluS NPs (a), then photobleached and casted with 50 μL of pure water (b) and with additional 50 μL of water containing 10 pM HA-RB observed after 60 min of incubation (c) or 50 μL of water containing 10 nM HA-RB observed after 5 min of incubation (d). (e) Plot of the intensity (log scale) with standard deviation in an area containing PluS NPs, after photobleaching and upon increasing the incubation time of 5 pM HA-RB or upon increasing the concentration of HA-RB from 5 pM to 5 nM (e). Wide-field image (f), BALM reconstruction (g), and their overlap (h) of a photobleached region of the coverglass, exposed to a drop of 5 pM HA-RB. (i) Plot of the intensity profiles of the wide-field image (green) and of the reconstructed image (red) across the three dotted lines indicated in (h). All scalebars are 5 μm; the excitation wavelength is 514 nm; TRITC microscope filter cube.

We performed a wide-field fluorescence microscope study, with single-molecule sensitivity, to deeply investigate the strength of the interaction taking place between HA-RB and PluS NPs and to assess the lower limit for this interaction to occur (and to be visualized). A coverglass was first functionalized with 3-aminopropyltriethoxysilane (APTES) and then with carboxylate-derivatized PluS NPs doped with rhodamine B dyes, via peptidic coupling (details in Supporting Information). Regions with islandlike surface coverage were identified on the coverglass and selected for the interaction study and super-resolution imaging, owing to the good contrast provided by the islands of PluS NPs compared with empty areas (Figure 5a). Addition of water on the coverglass (50 μL) resulted in no appreciable changes in the fluorescence of the monitored area, indicating that PluS NPs are firmly bound to the coverglass. The observation area was then photobleached down to reach almost zero signal from the PluS NPs (Figure 5b). After photobleaching, an additional drop of 50 μL of water was added (total volume = 100 μL) containing 10 pM HA-RB (in this way obtaining an on-site concentration of 5 pM, an extremely low value) and left to incubate for 5, 15, and 60 min. As a result, we observed clear local brightness enhancement on the islands of photobleached PluS NPs while only sparse emission events were collected from empty areas (Figure 5c,e). If a larger concentration is used, the signal of the PluS NP islands reaches immediately very high values, allowing fast imaging of the photobleached microislands (Figure 5d,e).

The rate of accumulation of HA-RB probe on the surface of PluS NPs can be pitted against its photobleaching rate to obtain distinguishable emission spots from individual interaction (binding) events. We obtained satisfying conditions to achieve an average of 30 localized emission spots per frame (ROI of 210 × 213 pixels), which in a series of 5000 frames provided about 150 000 binding-activated emission spots. The reconstructed map of the binding events shows a good match with the wide-field fluorescence image (Figure 5f–h) and enhanced resolution that becomes more evident where thinner islands are located, with full width at half-maximum (FWHM) reaching down to 150 nm (Figure 5h,i).

### Cell Membrane Synergic Internalization of HA-RB Nanogels with PluS Nanoparticles

**Figure 6:**
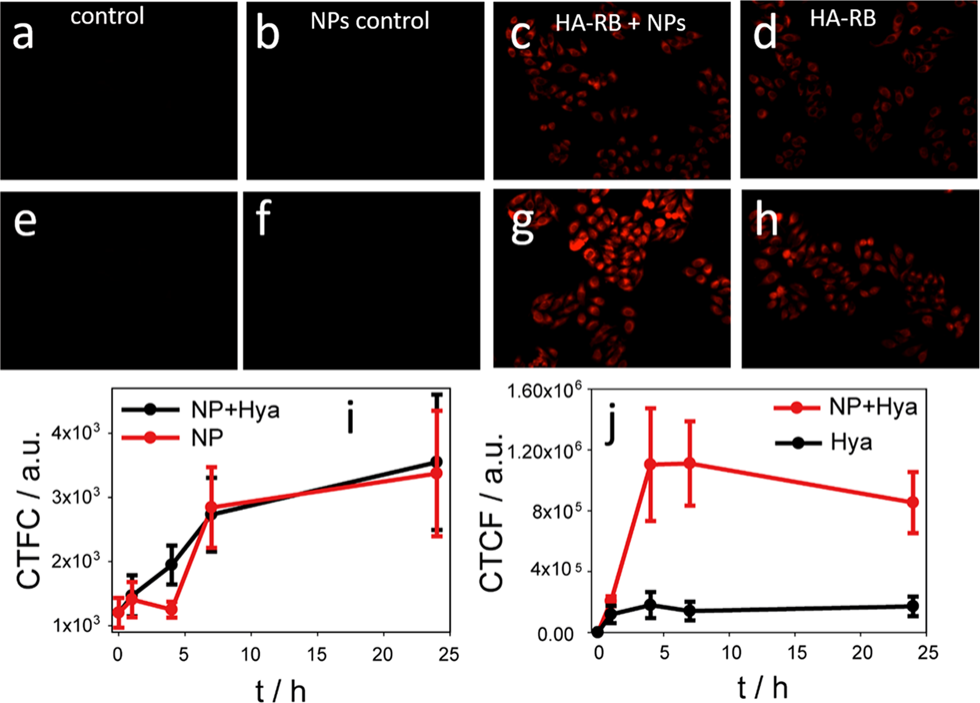
Fluorescence micrographs in the red channel of HeLa cells incubated with 1.5 μM HA-RB, with and without DEAC-doped PluS NPs (0.5 μM) for 1 h (a–d) and 24 h (e–h). (i–j) Corrected total cell fluorescence (CTCF) in the blue (DEAC-doped PluS NPs) and red (HA-RB) channels, respectively, of HeLa cells upon 0, 1, 4, 7, and 24 h of incubation.

Finally, we tested the ability of HA-RB, alone or in combination with PluS NPs, to be internalized into cells and to localize in specific compartments. We incubated HeLa cells with 1.5 μM HA-RB, in presence or absence of DEAC-doped PluS NPs (0.5 μM) and measured the intracellular emission signal of HA-RB and of DEAC NPs after 1, 4, 7, and 24 h of incubation (Figure 6a–h).

In presence of only PluS NPs (Figure 6b,f) or in the control (nor HA-RB or PluS NPs, Figure 6a,e), no emission can be observed in the RB emission region (red channel). In presence of only HA-RB, we observed a red emission rather homogeneously distributed within the cells, which increases in time in the first 4 h and then remains constant (Figure 6j). When cells are incubated with both HA-RB and PluS NPs (Figure 6c,g), the signal of HA-RB increases by more than 5 times compared with the case of HA-RB alone (Figure 6j). This large emission enhancement may be due either to an increase in the concentration of internalized HA-RB or to luminescence enhancement of HA-RB due to the interaction with PluS NPs. The blue channel signal, evidencing NPs internalization, does not reveal significant variations in the total intensity nor in the internalization kinetics, when NPs are incubated alone or in presence of HA-RB (Figure 6i).

**Figure 7:**
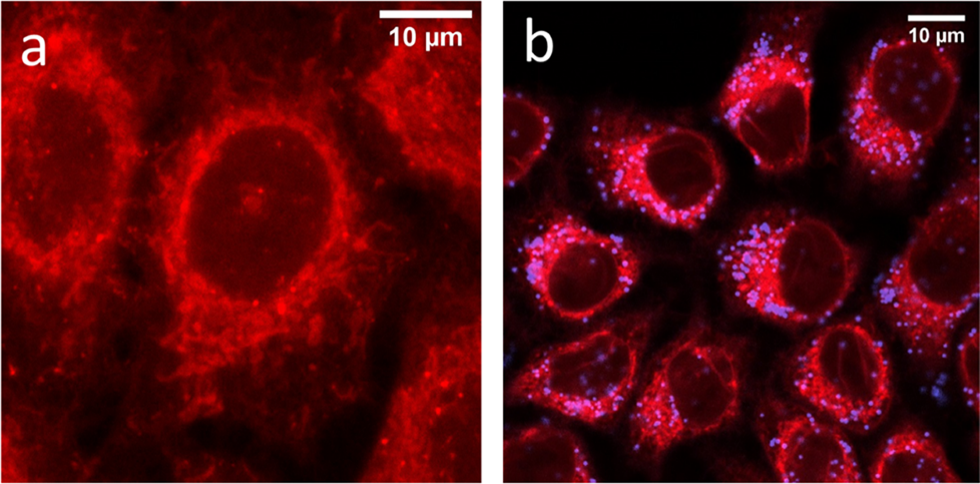
(a) Fluorescence confocal micrographs of HeLa cells incubated with 1.5 μM HA-RB for 24 h or (b) with 1.5 μM HA-RB and DEAC-doped PluS NPs (0.5 μM), also for 24 h (overlap of blue channel-DEAC emission), 401/460 ± 30 nm filter cube, and red channel-RB emission, 561/585 ± 20 nm filter cube.

Interestingly, the overlap of the red emission image (distribution of HA-RB, Figure S10) with the blue emission image (distribution of DEAC-doped PluS NPs, Figure S11) reveals that HA-RB and PluS NPs do not colocalize in cells (Figure 7a,b). While HA-RB is quite homogeneously distributed in cells as in absence of NPs, PluS NPs are mainly localized in the perinuclear region, where several membranous citoplasmatic networks (e.g., smooth and rough endoplasmatic reticulum, trans-Golgi) are usually located. In addition, we do not observe a significant enhancement of HA-RB emission in the regions where PluS NP signal is detected. Since the interaction between HA-RB and PluS NPs takes place at contact distances, these observations indicate that cellular uptake of HA-RB is more efficient in presence of PluS NPs, possibly due to the interaction with their surface, which partially disentangles the nanogel aggregates of HA-RB, facilitating HA-RB internalization by the cell. Nonetheless, once this interacting system enters the cell membrane, HA-RB spreads quite homogeneously in the cytoplasm, while NPs accumulate in the perinuclear region, according to a sort of “kiss and ride” mechanism. As demonstrated by comparing cell growth curves in the presence or in the absence of the nanomaterials (Figure S5), neither NPs nor HA-RB at the employed concentrations (0.5 μM NPs, 1.5 μM HA-RB) show any relevant effect on the cell viability. To better evaluate the fate of NPs, we acquired confocal images in the presence of lysotracker red after 24 h of incubation and no colocalization could be detected (Figure S6). The absence of colocalization with lysotracker indicates that the NPs are not segregated by lysosomes and, at least after 24 h, they are no longer retained.

This synergistic internalization with a subsequent differential fate might be employed to drive sophisticated drug delivery processes, with multiple addressable targets following the internalization of aggregates by the cells.

## Conclusions

In conclusion, we reported a simple protocol to synthesize hyaluronan nanogels doped with fluorescent dyes which, in water, are highly quenched. These nanogels show large luminescence enhancement upon interaction with nanomaterials and specifically with those bearing a soft and amphiphilic PEG-based shell, which are a large share of those applied in nanomedicine, because of the very favorable properties that PEGylation imparts to nanomaterials, including colloidal stability and prolonged circulation time. This high-affinity interaction that can be observed even at very low (picomolar) concentration allows to track these kinds of materials and to change their interactions with the environment, finally gaining some degree of control also on their lifecycle. In particular, two applications of broad interest for different and complementary communities are demonstrated herein: (i) a highly sensitive specific detection in an aqueous environment of PEGylated nanomaterials, offering an unreported tool for super-resolution microscopy and (ii) a synergistic mechanism for transport of HA nanogels and silica-based nanoparticles through cell membrane according to a kiss and ride mechanism. This rich chemistry at the nanoscale highlights the potential of HA-RB nanogels in monitoring the functions and the lifecycle of nanostructures in aqueous environments, as required for nanomedicine applications.

## Supporting Information Available

Materials and methods, including preparation and characterization of HA-RB and of all reported nanoparticles, protocols for BALM imaging, and cell internalization studies (PDF)

## Abbreviations

HA: hyaluronic acid
RB: rhodamine B
PEG: poly(ethylene glycol)
PluS: pluronic-silica
NPs: nanoparticles
FRET: Forster resonance energy transfer
FITC: fluorescein isothiocyanate
DEAC: diethylaminocoumarine
DMSO: dimethylsulfoxide
HAase: hyaluronidase
APTES: aminopropyltriethoxysilane
