# Supplementary material for: Specific, Surface-Driven, and High-Affinity Interactions of Fluorescent Hyaluronan with PEGylated Nanomaterials
Source: ACS Appl Mater Interfaces. 2020 Jan 29;12(6):6806–13. doi: 10.1021/acsami.9b17974 (PMC7993635; doi:10.1021/acsami.9b17974)
Supplement: Supplementary file 1 — am9b17974_si_001.pdf [file am9b17974_si_001.pdf]

Supporting Information

Specific, Surface-Driven and High-Affinity Interactions of Fluorescent Hyaluronan with PEGylated Nanomaterials

Francesco Palomba[a,§], Enrico Rampazzo[a], Nelsi Zaccheroni[a], Marco Malferrari[a], Stefania Rapino[a], Valentina Greco[b], Cristina Satriano[c], Damiano Genovese[a,]* and Luca Prodi[a,]*

a) Università di Bologna, Dipartimento di Chimica "Giacomo Ciamician", via Selmi 2, 40126, Bologna, Italy
b) Consorzio Interuniversitario di Ricerca in Chimica dei Metalli nei Sistemi Biologici (C.I.R.C.M.S.B.), via Celso Ulpiani, 27, 70125 Bari, Italy
c) Università degli Studi di Catania, Dipartimento di Scienze Chimiche, viale Andrea Doria 6, 95125 Catania, Italy

Corresponding Authors

*E-mail: damiano.genovese2@unibo.it

*E-mail: luca.prodi@unibo.it

Index

1. **Chemicals**
2. **Synthesis of PluS, MS and RM Silica Nanoparticles**
    a. **Pluronic Silica Nanoparticles Synthesis**
    b. **Mesoporous Silica Nanoparticles Synthesis**
    c. **Reverse Microemulsion Silica Nanoparticles Synthesis**
3. **Synthesis and characterization of functionalized hyaluronan**
    a. **Native Hyaluronic Acid (HA) characterization**
    b. **Functionalization of hyaluronan – synthesis of HA-RB**
4. **Characterization**
    a. **Photophysical measurements**
    b. **Atomic Force Microscopy (AFM)**
    c. **Dynamic Light Scattering (DLS) and Z-potential measurements**
    d. **TEM**
5. **BALM experiments**
    a. **Functionalization of coverglass with PluS NPs**
    b. **Images acquisition**
6. **Cell internalization experiments**

1. **Chemicals**: All reagents and solvents were used as received without further purification: Pluronic F127, tetraethylortosilicate (TEOS, 99.99%), chlorotrimethylsilane (TMSCl, ≥98%), Acetic acid (≥99.7%), cyclohexane, 1-hexanol, hexadecyltrimethylammonium bromide (CTAB), 7-(diethylamino) coumarin-3-carboxylic acid (DEAC, ≥ 98.0), 3-aminopropyltriethoxysilane (APTES) (≥98%), Fluorescein isothiocyanate (FITC) and Triton X-100 were purchased from Aldrich. Reagent grade NaCl was purchased from Fluka. A MilliQ Millipore system was used for the purification of water (resistivity <18 MΩ). Sodium hyaluronate (HA-190 kDa) was a kind gift from Fidia Farmaceutici S.p.A (IT).

2. **Synthesis of PluS, MS and RM Silica Nanoparticles**
   a. **Pluronic Silica Nanoparticles Synthesis (PluS)**: In a typical preparation of PluS nanoparticles, 100 mg of Pluronic F127 and the desired amount of the triethoxysilane derivative of DEAC (1.6 µmol, 0.2 % vs moles of TEOS, prepared as previously reported[1]) were carefully solubilized with 1.0 mL of dichloromethane in a 20 mL glass scintillation vial. The solvent was evaporated from the homogeneous solution by means of a gentle nitrogen flow and subsequently under vacuum at room temperature. NaCl (68 mg) was added to the solid residue, and the mixture was solubilized at 25 °C under magnetic stirring with 1.6 mL of acetic acid 1 M. TEOS (180 µL, 0.8 mmol) was then added to the resulting aqueous homogeneous solution followed by TMSCl (10 µL, 0.08 mmol) after 180 min. The mixture was kept under stirring for 48 h at 25 °C before dialysis treatments. The dialysis purification steps were carried out versus water on a precise amount of nanoparticle solution (1500 µL) finally diluted to a total volume of 10 mL with water, yielding a final PluS NPs concentration of 10 µM. [2] The morphology of PluS NPs was characterized with DLS and TEM, yielding the hydrodynamic diameter (d_H = 26 nm, polydispersity index PdI = 0.11) and the diameter of the silica core (d_C = 11 ± 1 nm), respectively.
   b. **Mesoporous Silica Nanoparticles Synthesis (MS)**: FITC functionalized mesoporous silica nanoparticles have been synthesized adapting a previously reported procedure.[3] Briefly, 0.911 g of CTABr and 0.617 g of Pluronic F-127 have been solubilized with 10 ml of HCl 0.01 M (pH 2) in a 25 ml round bottom flask. Under magnetic stirring 50 µL of a DMF solution of a triethoxysilane derivative of Fluorescein (obtained by reacting 0.025 mmol of FITC with 0.027 mmol of APTES in 250 µL DMF) and 1.08 mL of TEOS have been added. This resulting clear solution has been left to react for 24 h in the acidic media to promote the hydrolysis of the alkoxy silane. After that, 0.23 mL of ammonium hydroxide (28-30% wt) has been added to increase pH and promote the nucleation and condensation steps. After few

minutes the solution becomes turbid. After 2 h the nanoparticles are recovered by centrifugation at 5000 rpm and then washed by centrifugation 10 times with ethanol and 3 times with water to remove the excess of surfactants and to neutralize the medium.

c. **Reverse Microemulsion Silica Nanoparticles Synthesis (RM):** The synthesis of reverse microemulsion silica nanoparticles has been carried out adapting the procedure reported by Tan and coworkers. [4] The synthesis starts with the preparation of a water-in-oil emulsion, by mixing at room temperature, in a scintillation vial 1.83 g of Triton X-100, 7.5 mL of cyclohexane, 1.7 ml of 1-hexanol and 0.48 ml of deionized water. After 20 min 50 µL of the above described DMF solution of a triethoxysilane derivative of Fluorescein has been added followed by 100 uL of TEOS. Then 60 µL of ammonium hydroxide solution (28-30% wt.) has been added to the reaction. After 24h the nanoparticles are recovered by precipitation with 20 ml of acetone and then washed and centrifuged (5000 rpm, 10 min) several times with acetone, ethanol and finally with water.

3. Synthesis and characterization of functionalized hyaluronan

a. **Native Hyaluronic Acid (HA)**. Sodium Hyaluronate was analyzed by ^{1}H-NMR, CPG-SEC and viscosimetry analyses.

^{1}H-NMR spectra were recorded at 500 MHz on a Varian Unity Inova spectrometer. The experiments were performed in D_2O at 27 °C and the chemical shifts reported as δ (ppm), referenced to the resonance of residual HOD. Unequivocal assignments of ^{1}H resonances were supported by bi-dimensional experiment (COSY).

A high degree of chemical shift degeneracy, characteristic of high molecular weight hyaluronan polymer, [5] can be noted in the 1D ^{1}H-NMR spectrum of HA (**Figure S1**). Only three signals are resolved, i.e. those from the anomeric protons (GlcNAc H1 and GlcA H1) and from the methyl groups, which can be distinguished in the broad signals centered at δ = 4.55, 4.43 and 2.03 ppm, respectively. The chemical shift assignment of the remaining ten peaks is impaired by resonance overlap, since they are squeezed between δ = 3.90 and δ = 3.30 ppm. The 2D homonuclear experiments (Fig.S1, inset) confirm the correlation cross-peaks expected from literature spectra of HA. [SI-R5]

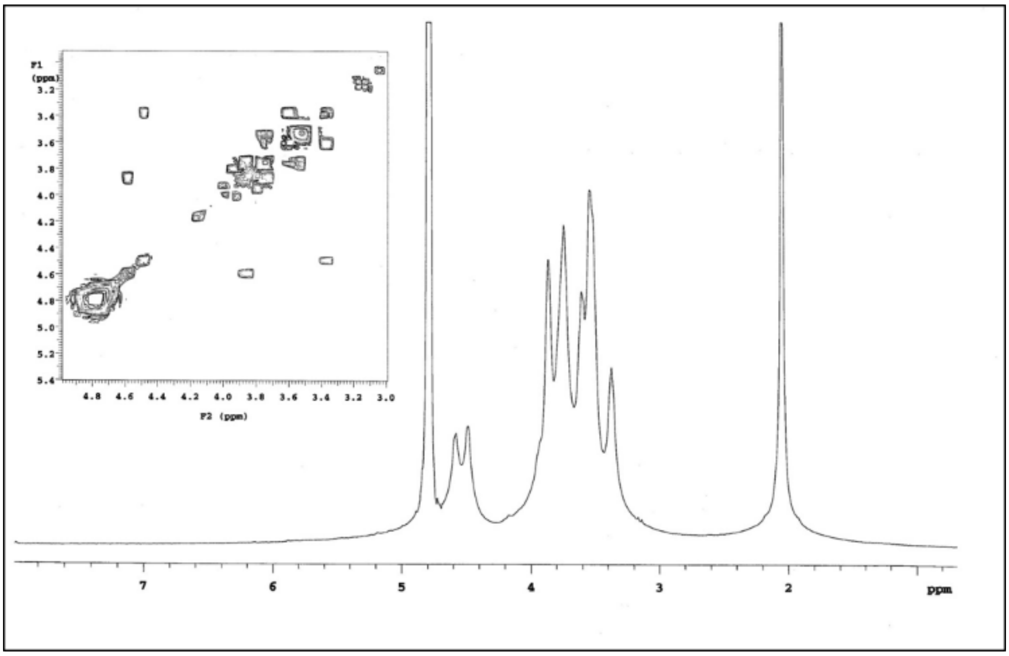

Figure S1. ^{1}H-NMR spectrum of HA in D_2O. Inset: COSY spectrum expanded from δ=5.0 to δ=3.0 ppm.

Measures of intrinsic viscosity and molecular weight distribution of HA were carried out on the chromatographic system GPCmax VE 2001 (Malvern), equipped with two TSK-GEL GMPWXL columns (7.8 mm ID×30 cm; Viscotek-TOSOH BIOSCIENCE) installed in series. The system is coupled to a sequence of three detectors: a refraction index detector, a light scattering detector and a four-capillary differential viscometer. The sample was eluted using a 0.1 M aqueous solution of sodium nitrate containing sodium azide (0.5 g/L) with a flow of 0.6 ml/min, at 40° C. The software Omnisec 4.1 was used for the acquisition and data analysis. A narrow distribution of molecular weights for the HA samples of 192.7 ± 2.0 KDa was found (with a standard deviation of about 10% of the measured MW values) and an intrinsic viscosity η = 5.2 ± 0.1 dL/g consistent with literature references for HA polymers with MW of the order of 2x10^5 Da. [6]

b. **Functionalization of hyaluronan – synthesis of HA-RB.** HA-RB nanogels are obtained via reaction of hyaluronic acid (193 ± 2 KDa) with Rhodamine B isothiocyanate in DMSO. In a typical preparation, 50 mg of hyaluronic acid 193 ± 2 KDa (0.26 μmol) were dispersed in 12 ml of DMSO in a scintillation vial and 9 mg of Rhodamine B isothiocyanate (16.8 μmol) were successively added under magnetic stirring. The reaction was left to proceed for 24 h at room temperature. The resulting dispersion was diluted with 36 ml of deionized water and dialyzed against water for at least three days.

4. Characterization techniques

a. **Photophysical measurements.** All NP solutions show very weak light scattering and can be treated from the photophysical point of view as any solution of molecular species. UV−vis absorption spectra were recorded at 25 °C by means of Perkin-Elmer Lambda 45 spectrophotometer. Quartz cuvettes with optical path length of 1 cm were used. The fluorescence spectra were recorded with an Edinburgh FLS920 equipped with a photomultiplier Hamamatsu R928P. The same instrument connected to a PCS900 PC card was used for the TCSPC experiments. Luminescence quantum yields (uncertainty, ±15%) were determined using rhodamine 101 solution in ethanol as a reference (Φ = 1.0). All fluorescence intensities were corrected for inner filter effects and reabsorption of emitted light according to standard methods. [7]

The effective dye doping degree was evaluated by UV-vis analysis measuring the absorbance of Rhodamine B, at known concentration of HA-RB, in ethanol solution, where the absorption spectrum features similar properties as the free dye, allowing us to use with a reasonable assumption the molar extinction coefficient of the free Rhodamine B dye in ethanol (106000 $M^{-1}cm^{-1}$).

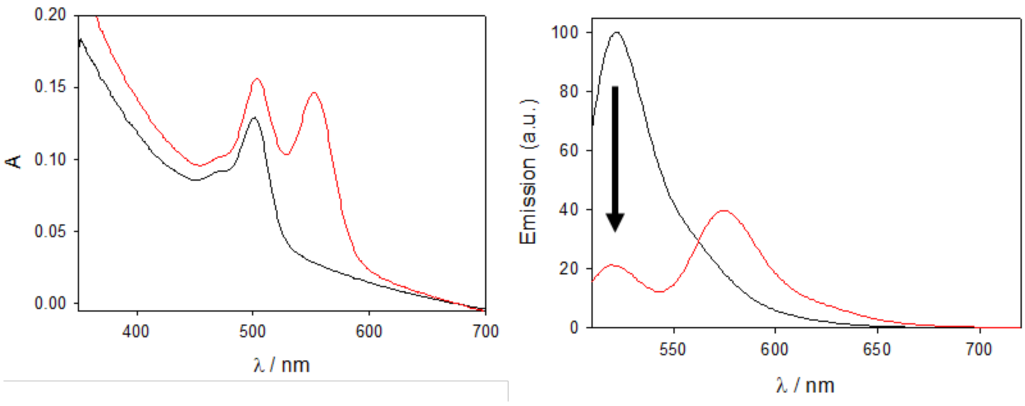

Figure S2 – Absorbance (left) and emission spectra (right, λ_{exc} = 500 nm) of mesoporous nanoparticles (0.055 mg/mL) before (black lines) and after addition of HA-RB (0.039 mg/mL, red lines), showing the quenching of fluorescein upon addition of HA.

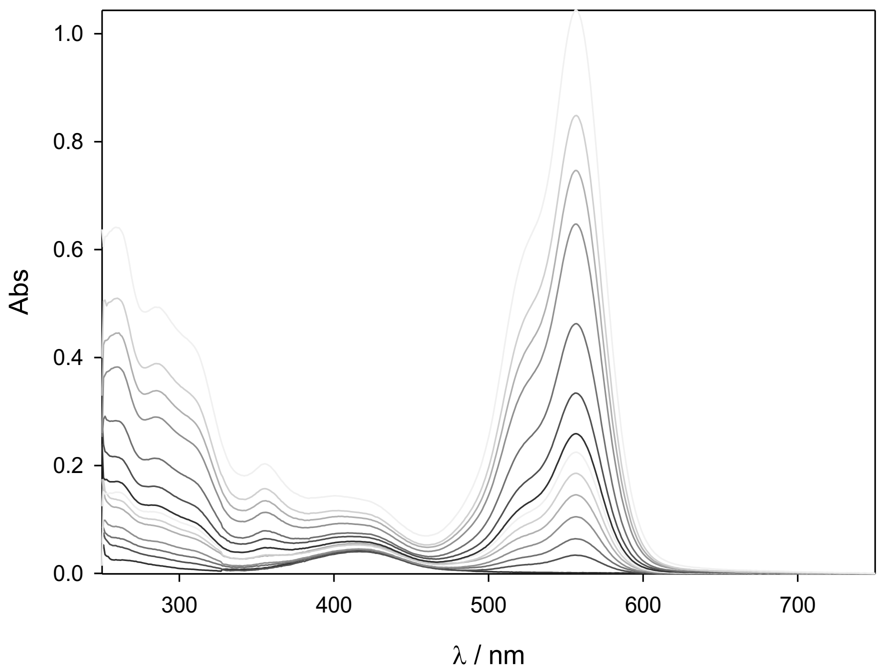

Figure S3 – Absorbance spectra of DEAC-doped PluS NPs (200 nM in water) titrated with increasing amounts of HA-RB (0-2 μM).

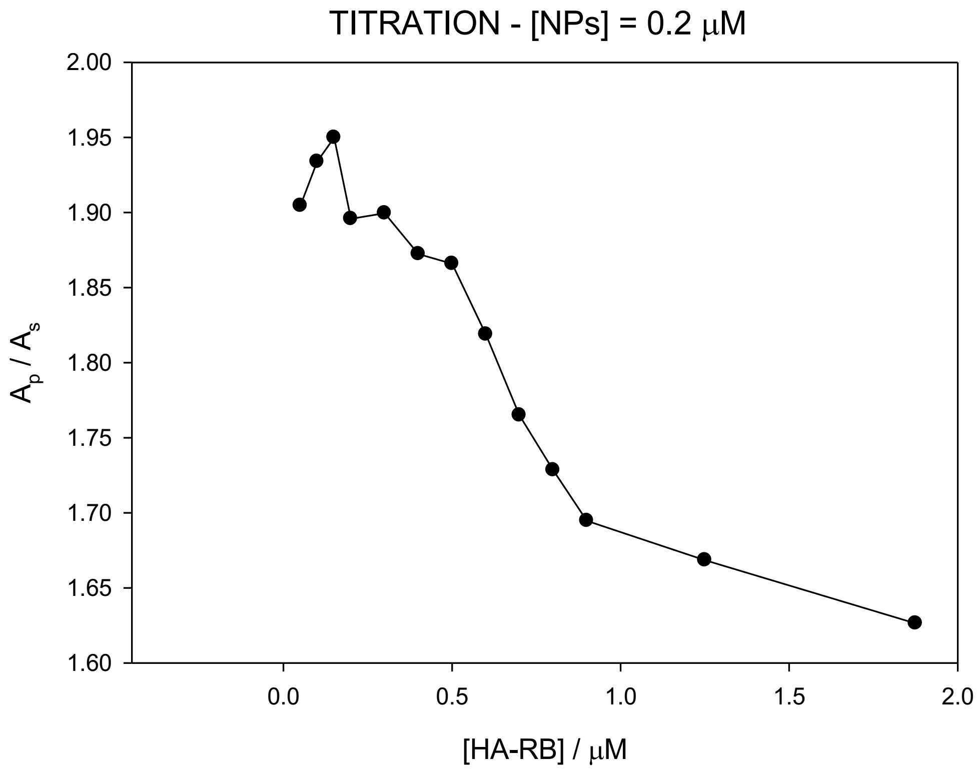

Figure S4 – Trend of A_p/A_s *vs* increasing amount of HA-RB in a solution of DEAC-doped PluS NPs 200 nM in water.

b. Atomic Force Microscopy (AFM). A Cypher AFM instrument (Asylum Research, Oxford Instruments, Santa Barbara, CA) operating in tapping AC-mode was used. For imaging in liquid, we used BioLever Mini BL-AC40TS (Olympus, Tokyo, Japan) probes with 30 kHz resonance frequency and 0.1 N m^1 spring constant (free oscillation amplitude of 20 nm; set point at ~70% of the free oscillation amplitude; scan rate 2.44 Hz). To image the samples, a 50 μL drop of the aqueous dispersion of PluS NPs ($2 \cdot 10^{-7}$ M), or Plus NP+HA-RB ($1 \cdot 10^{-7}$ M) was spotted on freshly cleaved muscovite mica (Ted Pella, Inc.). After 15 minutes of incubation at room temperature, samples were covered by 0.10 mL of ultrapure water and imaged. For imaging in air, tetrahedral silicon tips mounted on rectangular 30-μm long cantilevers (AT240TS Olympus) were used. The probes had nominal spring constants of 2 N/m and driving frequencies of 70 kHz. To image the samples, a 10 μL drop of the aqueous dispersion (50 nM in MilliQ water) of PluS NPs, HA-RB or Plus NP+HA-RB (1:1 mole ratio in the mixture) was put on freshly cleaved muscovite mica (Ted Pella, Inc.). After 5 minutes of incubation at room temperature, samples were washed with 1 mL of ultrapure water, dried under a gentle nitrogen stream and immediately imaged. Section analysis and particle size were measured using a free tool in the MFP-3DTM offline section analysis software.

c. DLS and Z-potential. The determination of the NP hydrodynamic diameter distributions was carried out through DLS measurements employing a Malvern Nano ZS instrument with a 633 nm laser diode. Samples were housed in disposable polystyrene cuvettes of 1 cm optical path length using water as solvent. The width of DLS hydrodynamic diameter distribution is indicated by PdI (polydispersion index). In the case of a monomodal distribution (Gaussian) calculated by means of cumulant analysis, PdI = $(\sigma/Z_{avg})^2$, where σ is the width of the distribution and Z_{avg} is average diameter of the particles population, respectively.

d. TEM. A Philips CM 100 TEM operating at 80 kV was used. For TEM investigations, a holey carbon foil supported on conventional copper microgrids was dried up under vacuum after deposition of a drop of NPs solution diluted with water (1:50). We obtained the size distribution by analyzing images with a block of several hundred NPs.

5. **BALM experiments**

a. **Functionalization of coverglass with PluS NPs.** The cover glass slides were treated with organic solvents (dichloromethane, ethanol), then with NaOH 1M and then they were immersed in ethanol. APTES (0.1 % v/v in ethanol) was added, and the functionalization was carried out overnight at room temperature. Coverglasses were repeatedly washed with ethanol and water, and then further immersed for 1 hour in a water solution of PluS NPs (20 nM) doped with rhodamine B and bearing carboxyl functional groups in the PEG terminations.[8] Finally, the coverglasses were carefully washed with MilliQ water several times, dried and used for microscopy experiments.

b. **Photobleaching of PluS NPs.** Regions of the coverglasses featuring a certain degree of inhomogeneous, island-like functionalization were chosen for an easier colocalization of PluS NPs and HA-RB. We used an Ar^+ ion laser (Melles Griot, IMA1 – Multiwavelength, 43 series ion laser) at 514 nm as the irradiation source (nominal power about 100 mW), at full power to bleach the signal from PluS NPs within 30 minutes of irradiation.

c. **Binding Activated Localization Microscopy Imaging.** A small drop (50-100 μL) of a water solution containing 5 pM HA-RB was deposited on the coverglass. The same laser line used for photobleaching was also used for imaging the binding events, at a reduced intensity (ND filter, ~10 mW residual transmitted power). The fluorescence emission is collected by means of an oil immersion objective (Olympus 100X, 1.3 NA) and sequences of 5000 frames are recorded onto a ROI of 210 x 213 pixel region (pixel size 160 nm) of an EMCCD camera (CCD Photon Max 512, Princeton Instruments) at a rate of 10 fps. The localization of single molecules is carried out using the free ImageJ plugin "QuicPALM". [9] The width of the features was determined by fitting selected profiles of intensity with a Gaussian function (f=y0+a*exp(-.5*((x-x0)/b)^2), where FWHM= 2.355*b).

6. **Cell internalization experiments**

Cell cultures, cellular uptake studies and fluorescence microscopy of DEAC-doped PluS NPs and HA-RB: HeLa cells were plated at 25000 cells/mL and grown overnight at 37°C and 5% CO_2 in DMEM (Dulbecco's Modified Eagle Medium; Thermo Fisher Scientific, 21969035) supplemented with 10% Fetal Bovine Serum (South America), 2 mM L-glutamine, 50 U/mL penicillin, 50 μg/mL streptomycin. After 24 hours cell culture medium was replaced with culture medium without phenol red containing 1.5 μM HA-RB or 1.5 μM HA-RB + 0.5 μM DEAC-doped PluS NPs and placed back in the incubator. For each time point, the compound rich medium was removed, and the cells were washed twice with phosphate-saline buffer

(PBS). Fluorescence images were acquired with a Nikon Eclipse Ti inverted microscope; HA-RB signals were measured with a Nikon Texas Red HYQ cubic filter (λ excitation = 532-587 nm, λ emission = 608-683 nm), while DEAC-doped PluS NP fluorescence was recorded by employing a Nikon BFP cubic filter (λ excitation = 379-401 nm, λ emission = 435-485 nm). Confocal images were acquired with a Nikon A1R confocal microscope, using two laser lines (401 and 561 nm) and two detection channels (460/30nm and 585/20nm) for the blue and red false colour channels, employed to measure the brightness of DEAC-doped PluS NPs and HA-RB (or lysotracker red), respectively. Lysotracker red was used to localize lysosomes and test colocalization with vesicles entrapping PluS NPs, by incubating HeLa cells with PluS NPs for 22 h and then adding lysotracker red for 2h.

7. Cell viability in the presence of in 0.5 μM NPs and in the presence of 0.5 μM NPs + 1.5 μM HA-RB.

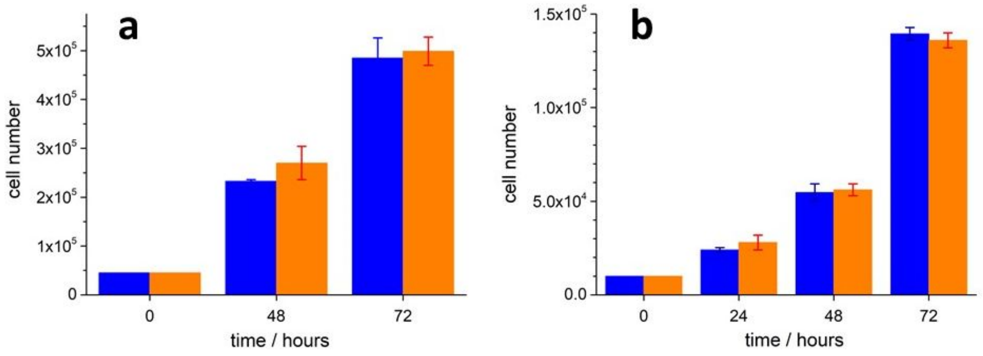

Figure S5 - Cell growth curves in the presence of 0.5 μM NPs (panel a) and 1.5 μM HA-RB + 0.5 μM NPs (panel b). Blue and orange bars represent data obtained in the absence and in the presence of the nanomaterials, respectively. Cells were incubated with the nanomaterials at 24 h (panel a) and 9 h (panel b) after plating; one standard deviation is reported as confidence interval.

8. Cell confocal images of Lysotracker + NPs.

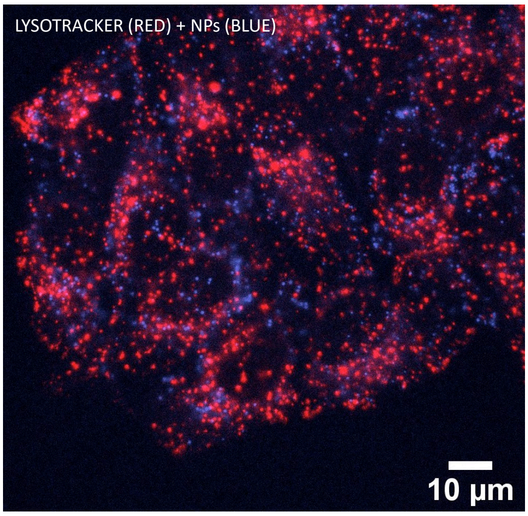

Figure S6 – Cell confocal images of Lysotracker + NPs after 24 hours of incubation.

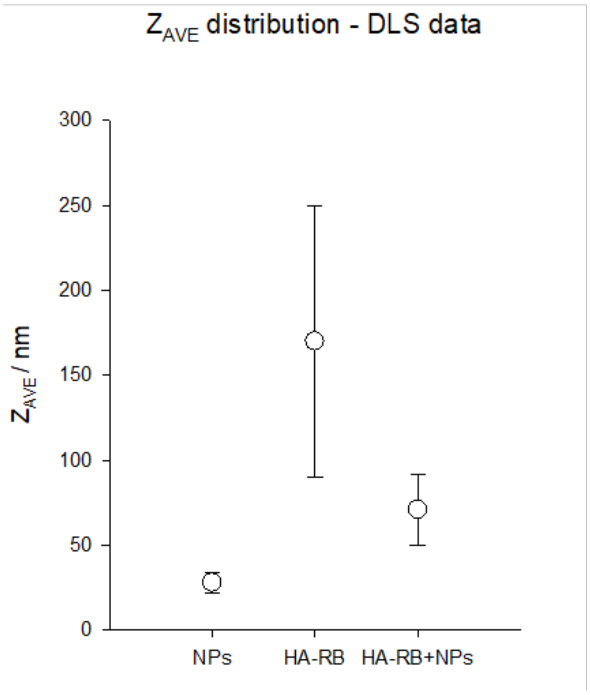

Figure S7 – Z-average distribution (with error bars) obtained with DLS measurements, for the starting systems (HA-RB and PluS NPs) and for the adduct (HA-RB+ PluS NPs).

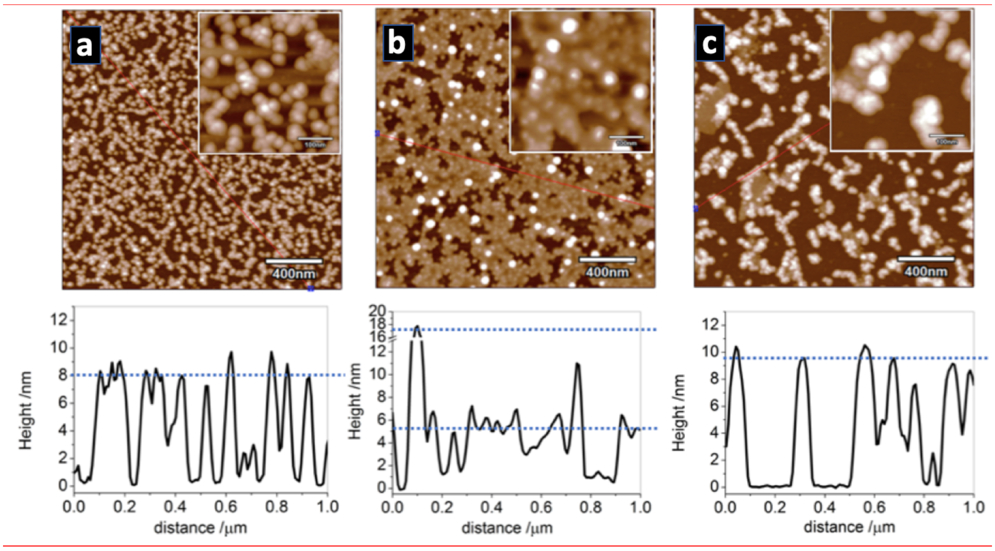

Figure S8 – AFM topography images recorded in AC mode in air and corresponding section analysis curves of: (a) PluS NPs, (b) HA-RB, (c) Plus NP+HA-RB (1:1 mole ratio). The z scale is 15 nm.

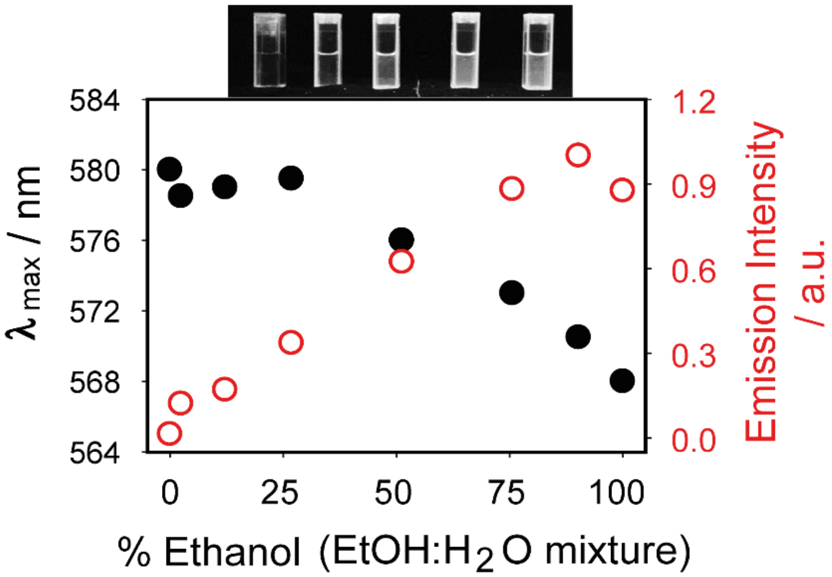

Figure S9 – Dependence of HA-RB photophysics (emission peak intensity and wavelength) on ethanol volume fraction in water:ethanol mixtures, and photograph of cuvettes (under UV 365 nm bench-lamp illumination) containing 200 nM HA-RB in ethanol:water mixture with 0, 20, 50, 80 and 100% ethanol, respectively.

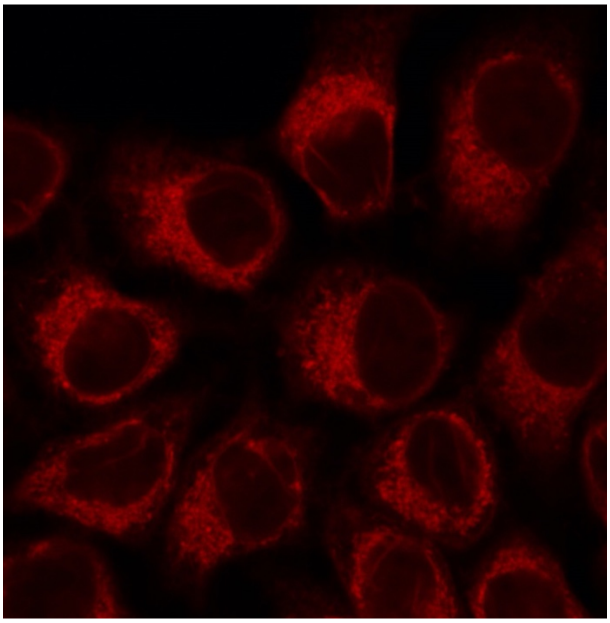

Figure S10 – Red channel of the confocal image shown in figure 7b.

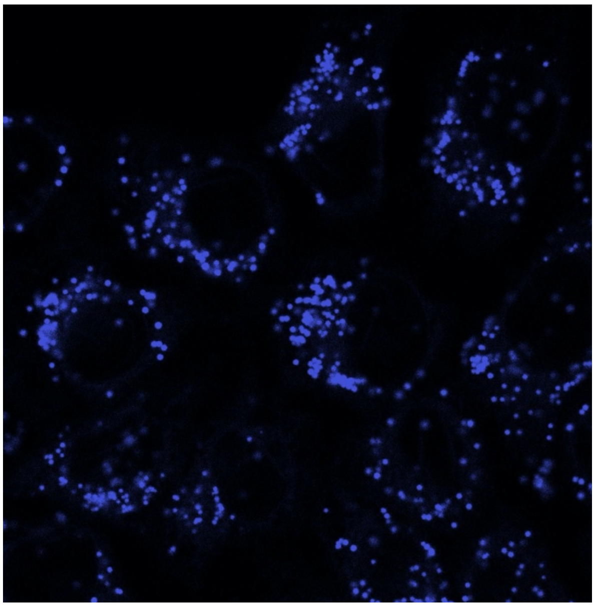

Figure S11 – Blue channel of the confocal image shown in figure 7b.

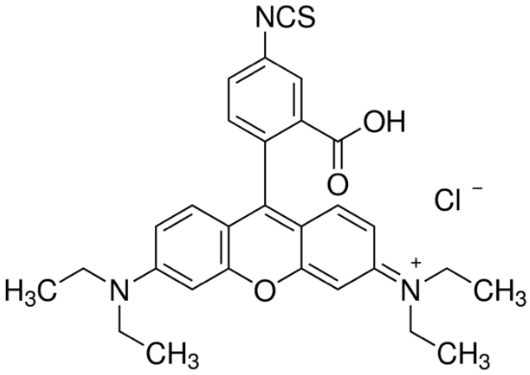

Figure S12 – Chemical formula of Rhodamine B isothiocyanate (RITC)

References

[1] E. Rampazzo, S. Bonacchi, D. Genovese, R. Juris, M. Montalti, V. Paterlini, N. Zaccheroni, C. Dumas-Verdes, G. Clavier, R. Meallet-Renault, L. Prodi, Silica Nanoparticles Doped with Multiple Dyes Featuring Highly Efficient Energy Transfer and Tunable Pseudo-Stokes-Shift , *J. Phys. Chem. C,* **2014**, *118*, 9261-9267

[2] E. Rampazzo, S. Bonacchi, R. Juris, M. Montalti, D. Genovese, N. Zaccheroni, L. Prodi, D. C. Rambaldi, A. Zattoni, P. Reschiglian, Energy Transfer from Silica Core-Surfactant Shell Nanoparticles to Hosted Molecular Fluorophores, *J. Phys. Chem. B,* **2010**, *114*, 14605-14613.

[3] K. Ikari, K. Suzuki, H. Imai, Structural Control of Mesoporous Silica Nanoparticles in a Binary Surfactant System, *Langmuir* **2006**, *22*, 802-806.

[4] R. P. Bagwe, L. R. Hilliard, W. Tan, Surface Modification of Silica Nanoparticles to Reduce Aggregation and Nonspecific Binding, *Langmuir* **2006**, *22*, 4357-4362.

[5] V. H. Pomin, NMR Chemical Shifts in Structural Biology of Glycosaminoglycans, *Anal. Chem.* **2014**, *86*, 65-94.

[6] E. Shimada, G. Matsumura, Viscosity and Molecular Weight of Hyaluronic Acids, *J. Biochem.* **1975**, *78*, 513-517.

[7] E. Rampazzo, R. Voltan, L. Petrizza, N. Zaccheroni, L. Prodi, F. Casciano, G. Zauli, P. Secchiero, Proper Design of Silica Nanoparticles Combine High Brightness, Lack of Cytotoxicity and Efficient Cell Endocytosis, *Nanoscale* **2013**, *5*, 7897-7905.

[8] M. Soster, R. Juris, S. Bonacchi, D. Genovese, M. Montalti, E. Rampazzo, N. Zaccheroni, P. Garagnani, F. Bussolino, L. Prodi, S. Marchio, Targeted Dual-Color Silica Nanoparticles Provide Univocal Identification of Micrometastases in Preclinical Models of Colorectal Cancer, *Int. J. Nanomedicine* **2012**, *7*, 4797-4807.

[9] R. Henriques, M. Lelek, E. F. Fornasiero, F. Valtorta, C. Zimmer, M. M. Mhlanga, QuickPALM: 3D Real-Time Photoactivation Nanoscopy Image Processing in Image, J *Nat. Methods* **2010**, *7*, 339-340.
